# Phase Response Synchronization in Neuronal Population with Time-Varying Coupling Strength

**DOI:** 10.1155/2015/816738

**Published:** 2015-11-10

**Authors:** Xianfa Jiao, Wanyu Zhao, Jinde Cao

**Affiliations:** ^1^School of Mathematics, Hefei University of Technology, Hefei 230009, China; ^2^Department of Mathematics, Southeast University, Nanjing 210096, China; ^3^Department of Mathematics, Faculty of Science, King Abdulaziz University, Jeddah 21589, Saudi Arabia

## Abstract

We present the dynamic model of global coupled neuronal population subject to external stimulus by the use of phase sensitivity function. We investigate the effect of time-varying coupling strength on the synchronized phase response of neural population subjected to external harmonic stimulus. For a time-periodic coupling strength, we found that the stimulus with increasing intensity or frequency can reinforce the phase response synchronization in neuronal population of the weakly coupled neural oscillators, and the neuronal population with stronger coupling strength has good adaptability to stimulus. When we consider the dynamics of coupling strength, we found that a strong stimulus can quickly cause the synchronization in the neuronal population, the degree of synchronization grows with the increasing stimulus intensity, and the period of synchronized oscillation induced by external stimulation is related to stimulus frequency.

## 1. Introduction

A nervous system's response to external stimulus can provide crucial information about its dynamical properties. The quantitative description of neuronal response to external stimulus has attracted great attention. The phase sensitivity function can quantitatively illuminate how an external stimulus affects the timing of spikes immediately after the stimulus in repetitively firing neurons, and it is also an important and effective method to study the dynamic behavior of synchronous activity in nervous system [1–5]. Moreover, neuronal synchronization plays a very important role in visual cortex [6], memory [7], and epilepsy [8]. It is known that neurons are coupled to each other via synapses and form neuronal networks. The synchronization of coupling neurons is the result of collective activity between neurons, which is considered as an essential mechanism to processing information in the neuronal population. To understand synchronized oscillation in the neuronal population, neurons can be modeled as neuronal oscillators. The Kuramoto model of coupled phase oscillators would provide a basis to modeling such synchronized oscillation [9–11]. In most studies, the Kuramoto models describe oscillators of fixed natural frequencies, fixed coupling strength. However, experimental studies have shown that synapses are plastic, that is, the coupling strengths among neurons can vary with time so that the neurons can instantly adjust their firing behavior and achieve new synchronization. Many complex behaviors induced by coupling types or coupling strengths have been found [12–16]. Moreover, rhythmic events are common in our sensory world; the biological rhythms constitute a natural forcing for neuronal oscillators. Hasselmo et al. have suggested that synaptic strengths onto pyramidal neurons from hippocampal region CA3 vary periodically with the theta cycle rhythm [17]. This intrinsic dynamics causes an additional periodic forcing [18]. Bîrzu and Krischer have investigated the dynamics of a population of globally coupled FitzHugh-Nagumo oscillators with a time-periodic coupling strength and have observed rich oscillatory and resonant behavior [19]. The multiple coherence resonances induced by the time-periodic coupling strength have been observed in scale-free networks of bursting neurons [20]. The brain permanently receives natural sensory stimulation, whereas experimental electrical or magnetic stimulation of the nervous system is used for analyzing the dynamical interaction of different brain areas. Therefore, it is of great importance to understand how a stimulus influences synchronized neuronal activity. The effects of periodic stimuli on rhythmic biological activity were experimentally studied in a variety of physiological paradigms. Mathewson et al. have reported that rhythmic visual stimuli can entrain ongoing neural oscillations in humans [21]. Will and Berg have found that periodic auditory stimulation can produce brainwave synchronizations that are likely to affect various cognitive functions [22]. In present paper, we investigate the effect of time-periodic coupling strength on the synchronized behaviors of neuronal population subjected to external harmonic stimulus by the use of phase sensitivity function.

The stimulation paradigm leads to a persistent increase of the synaptic transmission efficacy; the effect is called long-term potentiation of synapses. Long-term potentiation (LTP) is an important form of the synaptic plasticity [23] and is an important mechanism to learning and memory. LTP affects the transmission of information and coupling strength between neurons in the presence of stimulus, thus affecting the efficiency of synaptic learning. In this paper, based on phase response model of population of coupled neuronal oscillators, we consider the dynamics of the coupling strength in order to explore the effect of external stimulus on synchronized oscillation in neuronal population.

## 2. Model Equations 

Neuronal synchronization in the brain has often been investigated by Kuramoto model [9–11]

(1) $$\begin{array}{c} {\dot{\theta }}_{i}=\omega_{i}+\frac{K}{N}\overset{N}{\underset{i=1}{\sum }}\sin\left(\;\theta_{j}-\theta_{i}\;\right)\;i=1,2,\ldots ,N. \end{array}$$

Reference [9] describes how synchrony occurs when *K* is above a critical value.

*θ*_*i*_ is the phase of the *i*th oscillator, *w*_*i*_ is the eigenfrequency of *i*th oscillator, *K* is the synaptic coupling strength between neuronal oscillators, and (*θ*_*j*_ − *θ*_*i*_) is the difference in phases between the *i*th and *j*th neuronal oscillators.

We consider a population of *N* globally coupled neuronal oscillators subject to harmonic stimulus; the dynamical equations is as follows:

(2) θ˙i=wi+1N∑i=1NKsin⁡θj−θi+Ftsin⁡θi,for  i=1,2,…,N,N>1,

where *F*(*t*) is an external harmonic stimulus; sin⁡*θ*_*i*_ is a phase sensitivity function.

In order to study the synchronized behavior of neuronal population, we introduce time-varying order parameter as [9]

(3) $$\begin{array}{c} z\left(\;t\;\right)=r\left(\;t\;\right)e^{i\phi \left(t\right)}=\frac{1}{N}\overset{N}{\underset{j=1}{\sum }}e^{i\theta_{j}}, \end{array}$$

where *r*(*t*) and *ϕ*(*t*) measure time-varying average amplitude and phase, respectively, *r*(*t*) describes synchronization degree of neuronal population, 0 ≤ *r*(*t*) ≤ 1, and the higher *r*(*t*) indicates the stronger synchronization.

Substituting (3) into (2), it yields

(4) $$\begin{array}{c} {\dot{\theta }}_{i}=w_{i}+Kr\left(\;t\;\right)\sin\left(\;\phi \left(\;t\;\right)-\theta_{i}\;\right)+F\left(\;t\;\right)\sin\theta_{i}, \end{array}$$

when *N* → *∞*, as in [9], (3) turns into

(5) ∂f∂t+∂∂θw+K2ize−iθ−z∗eiθ+Fteiθ−e−iθ2if=0;

*f*(*w*, *θ*, *t*) is continuous distribution function of phase *θ* with natural frequency *w*, that is, a probability distribution function (PDF) with phase *θ* at time *t*; *∗* represents the complex conjugate. Therefore, the arithmetic mean value of (3) becomes the average of the phase and frequency; namely,

(6) $$\begin{array}{c} z=\overset{+\infty }{\underset{-\infty }{\int }}\overset{2\pi }{\underset{0}{\int }}g\left(\;w\;\right)f\left(\;\theta ,w,t\;\right)e^{i\theta }d\theta \;dw. \end{array}$$

The normalized PDF is

(7) $$\begin{array}{c} \overset{2\pi }{\underset{0}{\int }}f\left(\;\theta ,w,t\;\right)d\theta =1. \end{array}$$

In the dynamic systems, *f*(*θ*, *w*, *t*) is 2*π*-period function, so it can be expanded as Fourier model. In general, in order to simplify (6), we apply the Ott and Antonsen ansatz [24]:

(8) $$\begin{array}{c} f\left(\;\theta ,w,t\;\right)=\frac{1}{2\pi }\left\{\;1+\left\{\;\overset{\infty }{\underset{n=1}{\sum }}{\left[\;\alpha \left(\;w,t\;\right)\;\right]}^{n}e^{in\theta }+\text{c.c.}\;\right\}\;\right\}, \end{array}$$

where c.c. denotes complex conjugate. Substituting (6) into (4), we obtain an evolution equation about *α*:

(9) $$\begin{array}{c} \frac{\partial \alpha }{\partial t}+iw\alpha +\frac{K}{2}\left(\;z\alpha^{2}-z^{*}\;\right)+F\left(\;t\;\right)\frac{1-\alpha^{2}}{2}=0. \end{array}$$

The order parameter in (5) can be written as

(10) $$\begin{array}{c} z^{*}=\overset{+\infty }{\underset{-\infty }{\int }}\alpha \left(\;w,t\;\right)g\left(\;w\;\right)dw. \end{array}$$

*α*(*w*, *t*) in the complex plane is continuous, and natural frequency *w* in (10) follows a Lorentzian distribution *g*(*w*), which can be written as [25]

(11) $$\begin{array}{c} g\left(\;w\;\right)=\frac{1}{\pi \left(w-w_{0}+\gamma i\right)\left(w-w_{0}-\gamma i\right)}. \end{array}$$

*γ* is the scale parameter which specifies the half-width at half-maximum; *w*_0_ is the mean of *g*(*w*). According to (10), the residue at the pole *ω* = *ω*_0_ − *γi* as *z* ^*∗*^ = *α*(*w*_0_ − *γi*, *t*), which is applied in (9), so we can obtain

(12) $$\begin{array}{c} \frac{\partial z}{\partial t}=i\omega_{0}z-z\left[\;\gamma +\frac{K}{2}\left(\;{\left|\;z\;\right|}^{2}-1\;\right)\;\right]-\frac{F}{2}\left(\;1-z^{2}\;\right). \end{array}$$

Substituting (3) into (12), we finally obtain

(13) drdt=−rγ+K2r2−1−Ft21−r2cos⁡ϕ,dϕdt=w0+F21r+rsin⁡ϕ.

## 3. Numerical Simulations

**Figure 1:**
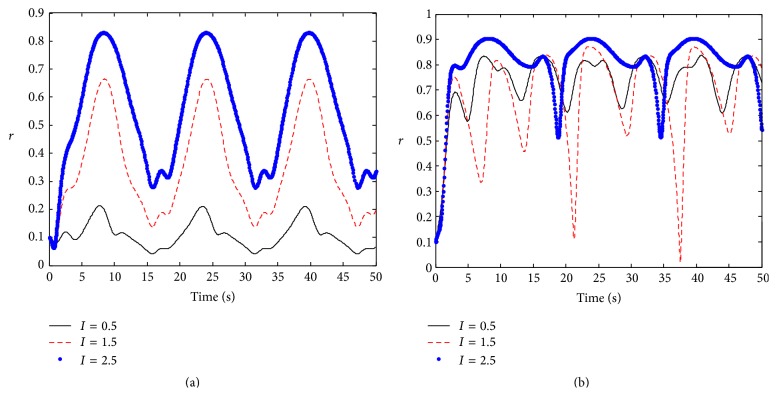
The evolution of the amplitude of the order parameter with respect to time in time-periodic coupling strength. Parameters: *γ* = 0.5, *Ω* = 0.8, *w*_0_ = 1.5, *ε* = 0.8, and  *c* = 0.2; (a) *k* = 0.05, and (b) *k* = 2.5.

**Figure 2:**
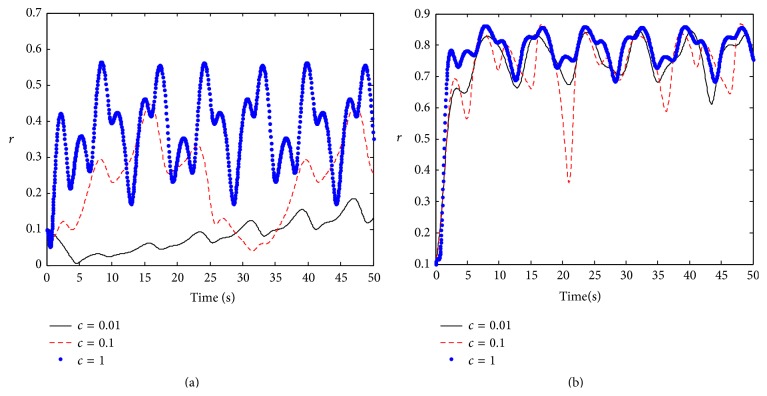
The evolution of the amplitude of the order parameter with respect to time in time-periodic coupling strength. Parameters: *γ* = 0.5, *Ω* = 0.8, *w*_0_ = 1.5, *ε* = 0.8, and  *I* = 1; (a) *k* = 0.05, (b) *k* = 2.5.

As the natural frequency of a neuronal oscillator subject to a Lorentzian distribution, we investigate the effect of time-varying coupling strength on the phase response synchronization. Let *F*(*t*) = *I*sin⁡(*ct*); here *I* is stimulus intensity; *c* is stimulus frequency. We first consider time-periodic coupling strength *K*(*t*) = *k* + *ε*cos⁡(*Ωt*), where *ε* is the amplitude and *Ω* is the frequency of the time-periodic coupling strength; *k* is the inherent coupling strength between neuronal oscillators. When the coupling is weak (e.g., *k* = 0.05), a stimulation with small intensity can not cause significant synchronized activity, but there is a periodic synchronization in the neuronal population with strong stimulation; the stronger stimulation leads to the higher degree of synchronization (Figure 1(a)). When the coupling is strong (e.g., *k* = 2.5), an external stimulus always causes periodically synchronized activity regardless of whether the stimulus intensity is weak or strong (Figure 1(b)). To understand the dependence of the synchronization in the neuronal population on the stimulation frequency, we also compute the amplitude of the order parameter in Figure 2. When the coupling is weak (e.g., *k* = 0.05), there is no synchronization in the neuronal population for low-frequency stimulation. However, when the stimulation frequency increases, the neuronal population becomes periodically synchronized oscillation, and the oscillatory frequency is higher for the higher stimulation frequency (Figure 2(a)). When the coupling is strong (e.g., *k* = 2.5), the variation of stimulus frequency has little influence on synchronization behavior (Figure 2(b)). This shows that the neuronal population with stronger coupling strength has good adaptability to stimulus.

We also consider the dynamics of coupling strength $\dot{K}=-\tau K+D\sum_{k}^{}\delta \left(t-t_{k}\right)$, *τ* is the attenuation constant of coupling strength, *D*∑_*k*_ *δ*(*t* − *t*_*k*_) is the spiking input, which modulates synaptic coupling strength, and *D* is a constant parameter that determines the amplitude of the postsynaptic response to an incoming spiking.

**Figure 3:**
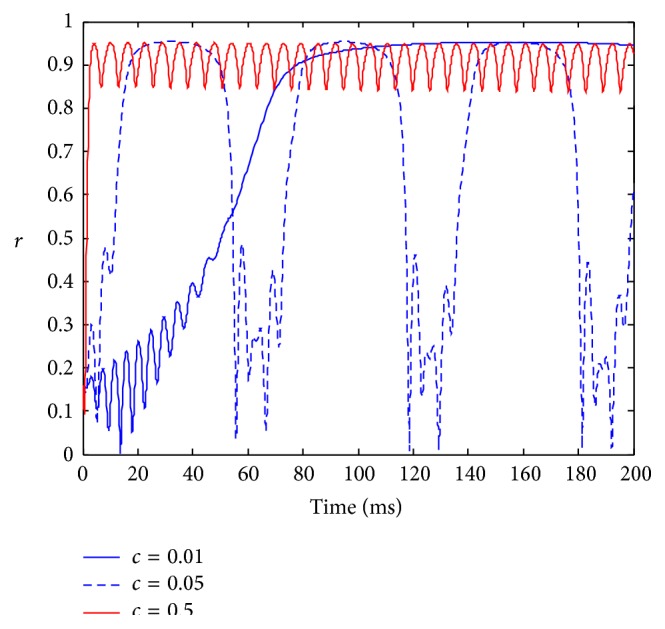
The evolution of the amplitude of the order parameter with respect to time in the presence of stimulation with different frequency. Parameters: *I* = 5, *γ* = 0.1, *w*_0_ = 1.5, *τ* = 0.005, and *D* = 0.001.

The evolution of the amplitudes of the order parameter of the neuronal population for different frequency stimulation is shown in Figure 3. As the stimulation strength is moderate (e.g., *I* = 5), the low-frequency stimulation can cause long-term complete phase synchronization in the neuronal population. However, when the stimulation frequency increases, the neuronal population has a quick response to a high-frequency stimulus, and the synchronization becomes periodic oscillation. The high-frequency stimulation can induce high-frequency synchronized oscillation. This shows that the frequency of synchronized oscillation depends on the stimulation frequency.

**Figure 4:**
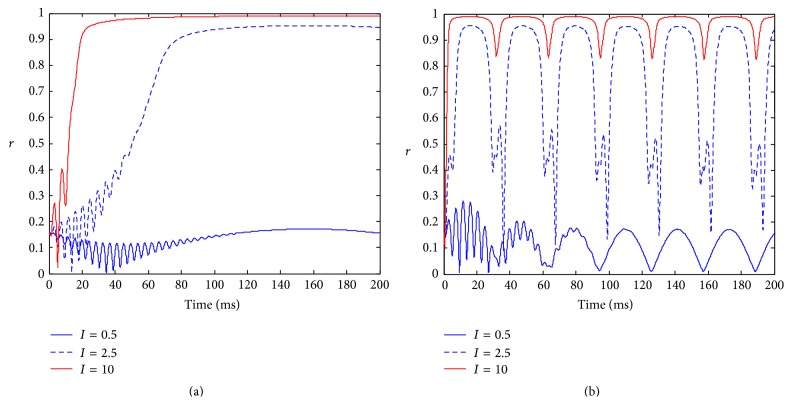
The evolution of the amplitude of the order parameter with respect to time in the presence of stimulation with different intensity. Parameters: *γ* = 0.1, *w*_0_ = 1.5, *τ* = 0.005, and  *D* = 0.001; (a) *c* = 0.01, (b) *c* = 0.1.

Figure 4 illustrates the evolution of the order parameter for different intensity stimulations. When the stimulation frequency is low, there is no synchronized activity in the neuronal population for a weak stimulation (Figure 4(a)). However, when the stimulation intensity increases, the complete synchronization in the neuronal population is observed, and the stronger stimulation quickly causes phase synchronization (Figure 4(a)). This means that the synchronized response time is related to the stimulation intensity in neuronal population for a low stimulation frequency. For higher frequency stimulation, a weak stimulation can only cause low synchronized oscillation; as the stimulation intensity increases, periodically synchronized oscillation occurs in the neuronal population, and there is an explicit tendency that the degree of synchronization increases when the stimulation intensity increases. Moreover, there is no change of the frequency of the synchronized oscillation when the stimulation intensities are deferent (Figure 4(b)). This result shows that the degree of synchronization can be used to encode the information of the stimulation intensity for high-frequency stimulation.

## 4. Conclusions

The synchronization of oscillatory neuronal activity is a fundamental mechanism for combining related neuronal information. The dynamic models of globally coupled phase oscillators have been proposed for the study of neuronal synchronization in the brain [10–14]. In the brain, the synaptic coupling between neurons is plastic. The dynamics of globally coupled neuronal oscillators with varying coupling strengths have been investigated in recent years [15, 16, 18–20]. The effect of external stimulation is often not considered in the studies mentioned above. We suggested that the quantitative description of neuronal response to external stimulation is of great importance in understanding neuronal dynamics in the presence of external stimulation. Therefore, the phase sensitivity function is used to describe neuronal response. The dynamic model of globally coupled neuronal population with time-varying coupling strengths was developed by introducing phase sensitivity function in the presence of external stimulation. The synaptic strengths vary periodically with the biological rhythms; we assumed that the coupling strength is time-periodic. Numerical simulations have shown that when the coupling is weak, the periodically synchronized oscillation is induced by the external stimulation with stronger intensity or higher stimulation frequency. When we considered long-term potentiation in the synaptic transmission efficacy, the synaptic dynamics was introduced in the model. We have found that the synchronized response time is related to the stimulation intensity. Our results suggest the stimulation intensity is relevant for the degree of synchronization; the stimulation frequency is relevant for the frequency of synchronized oscillation in the neuronal population.
